# A new screening index to better target low-level lead exposure in Atlanta, Georgia

**DOI:** 10.1038/s41598-020-75000-0

**Published:** 2020-10-22

**Authors:** Samantha Distler, Eri Saikawa

**Affiliations:** 1 Department of Quantitative Theory and Methods, Emory University, Atlanta, GA 30322 USA; 2 Present Address: Department of Epidemiology, School of Public Health, University of Michigan, Ann Arbor, MI USA; 3 Department of Environmental Sciences, Emory University, Atlanta, GA 30322 USA

**Keywords:** Environmental impact, Risk factors

## Abstract

Lead poisoning is often seen as a problem of the past. While acute cases are now rare, there is no known safe level of lead for children and blood lead levels at and below 5 μg/dL are associated with neurological deficits. Previous work has established that risk factors for lead exposure include race/ethnicity, poverty, Medicaid enrollment, housing built before 1950, and age. Efficient blood lead screening is crucial in the greater Atlanta area as pockets of poverty and old housing put some children at particularly high risk for chronic exposure to low levels of lead. Here, 20 years of data on children’s blood lead levels in Georgia were used to create maps to assess the spatial distribution of blood lead screening and blood lead levels in the Atlanta area. ZIP code tabulation area (ZCTA)-level screening rates continue to be associated with relative poverty but not with housing age, a well-established risk factor for lead exposure. Building on previous research, a priority screening index based on poverty and housing age was also created to identify specific high-risk census tracts for screening within Atlanta ZCTAs. This index shows a total of 18 highest-priority census tracts in the greater Atlanta area. Together, these 18 tracts contain 2715 children under six years old, 1.7% of all children under six years old in the entire greater Atlanta area.

## Introduction

Lead, one of the oldest known toxicants, affects every organ system in the human body^1,2^. Many of the most concerning effects of lead are neurological. High (60 to 300 μg/dL) blood lead levels (BLLs) are associated with severe neurologic outcomes like peripheral neuropathy and encephalopathy with symptoms that include ataxia, convulsions, coma, and death^1,2^. Such severe cases are now rare in the United States (US) due to federal regulations that reduced lead in paint, solder, gasoline, and other common exposure sources^3–5^. However, ongoing work continues to indicate that far lower BLLs are also associated with negative health concerns. Low-level lead exposure has been linked to immunological and endocrine effects, as well as cardiovascular disease, a major cause of adult mortality in the US^6,7^. Neurologically, lower BLLs are associated with attention deficit hyperactivity disorder (ADHD), impulsivity, cognitive deficits, IQ decrements, and neuromotor changes^1,2^.

Lead poisoning was only clinically defined until the Surgeon General’s 1971 report stated that a BLL of 40 μg/dL indicated “undue absorption” of lead^8^. In the following decades, this threshold was repeatedly lowered until a 1991 Centers for Disease Control and Prevention (CDC) policy defined the BLL of concern as 10 μg/dL^9^. In 2012 the CDC moved away from a “level of concern” in favor of a “reference value” based on the 97.5th percentile BLL in children between 1–5 years^6^. This blood lead reference value was calculated as 5 μg/dL based on National Health and Nutrition Examination Survey (NHANES) data from 2007 to 2010^10^. One major reason for switching from a static threshold level to a relative reference value was to reflect the now widely-accepted idea that there is no known safe level of lead for children.

Young children are most likely to ingest lead due to their physical proximity to surface dust and behaviors like crawling and hand-to-mouth activity^1,11^. Once ingested, children also absorb up to five to ten times more lead in the gastrointestinal tract than adults^12–14^. Absorbed lead then enters children’s and infants’ developing nervous systems which is particularly vulnerable in part because the blood–brain barrier is not fully formed^15,16^. Lead is known to disrupt processes like neuronal migration and synapse pruning that are crucial for brain development^1,2,17^.

Decades of research have shown that some children have an even higher risk of lead exposure than others^18,19^. Living in older housing is a risk factor for lead exposure because of the possible presence of lead-based paints. Residential paints containing more than 0.06% lead were banned in 1978^20^. While all homes built before 1978 have an increased risk for lead exposure, houses built before 1950 pose the greatest danger as they are more likely to contain lead-based paint and that paint is more likely to contain higher levels of lead^21^. Certain demographic groups are also at an increased risk for lead exposure due to lack of resources and a long history of American environmental injustice. Socioeconomic factors associated with an increased risk for lead exposure include poverty, race/ethnicity (specifically, being non-Hispanic black), and Medicaid coverage^5, 22–24^. Bernard & McGeehin^25^ found that these well-documented socioeconomic risk factors and housing age continue to be associated with elevated BLLs even when the newer, lower BLL of 5 μg/dL is used as a reference value.

When they proposed using a reference value the CDC’s Advisory Committee on Childhood Lead Poisoning Prevention (ACCLPP) stressed the importance of primary prevention and reducing disparities based on housing, race/ethnicity, and socioeconomic factors^6^. The CDC has recommended targeting high risk children and neighborhoods since 1997^21^. Now, given the overall decline in BLLs in the larger US population, effectively targeting high-risk communities is more important than ever to reach those at risk for subtler chronic exposures.

Several papers have assessed methods for targeting high risk children and locations. Some researchers have focused on building regression models to predict BLLs based on demographic characteristics, such as race, age, Medicaid coverage, and income^5,22,26^. Other targeting methods stress the use of geographic information systems (GIS) to create maps highlighting areas with high proportions of older homes and/or elevated BLLs^27,28^. Different studies have assessed lead exposure at the level of counties, ZIP codes, census tracts, communities, block groups, tax parcels, and neighborhoods^22,27–34^. Smaller geographic areas may be able to better explain BLLs because they capture a more specific area. Research has indicated that census tracts and census block groups better explain BLLs than ZIP codes^26,35^. When risk is only assessed for large areas, aggregate values can obscure pockets of high risk.

In Atlanta, Georgia, the presence of risk factors, such as older housing, poverty, and high-density urban areas put some children at risk for lead exposure. Vaidyanathan et al.^36^ developed a geospatial strategy to assess Atlanta lead screening rates and results by neighborhoods since these areas are both small and easily recognizable. Using data from 2005, the authors created a priority screening index of Atlanta neighborhoods using housing age and enrollment in Georgia’s Special Supplemental Nutrition Program for Women, Infants, and Children (WIC), a proxy for poverty. To indicate the urgency of lead screening, this index assigns every geographic area a score from 2 to 8 based on housing age and WIC quantile breaks. The authors also analyzed existing screening rates and found that neighborhood blood lead testing did rise with increasing WIC enrollment but was not significantly associated with the proportion of houses built before 1950 or 1978.

The Georgia Department of Public Health (DPH) recommends that all children enrolled in Medicaid receive blood lead tests at age one and two or anytime between ages three and six if no record of previous testing exists^37^. Furthermore, the most recent guidelines state that DPH is focused on screening children who are in a high-risk group (such as WIC enrollment) or geographic location. For high-risk geographic locations, the DPH website lists 14 counties where children “may have a higher risk of lead exposure”^37^.

These counties are the 14 high-risk counties identified in Rustin et al.^38^. In this paper the authors used GIS to create maps of Georgia based on housing age and occupancy type (owning vs renting), two components of housing-based lead exposure risk. However, the authors do not use this housing information to label counties as high risk. Instead, the raw number of children with BLLs above 5 μg/dL in 2013 was used to identify the 14 counties. Thus, many of these counties are those with the most children under six years old and/or the most children screened. The authors note that the map highlighting these 14 counties, an updated version of a similar map of 2010 data, is meant to be added to the housing age maps. However, so far no combined map exists^37^.

The overwhelming drop in children’s BLLs in the US is arguably one of the greatest public health achievements in the nation’s history^39^. In order to continue to further that progress in this new era of lower-level chronic exposures, state and local authorities must be able to identify high-risk areas. Despite characteristics of the Atlanta area putting children at potentially high risk for lead exposure, there is no simple, replicable system for using available data to identify specific high-risk areas and target them for blood lead screening. Here, twenty years of data from the DPH and the US Census Bureau is used to understand children’s BLLs and lead screening in the greater Atlanta area and construct a priority screening index to direct targeted testing.

## Methods

All BLL data used here come from DPH and refer to children under six years old. ZIP code-level data were requested through the DPH Public Health Information Portal while county level data were retrieved from reports published on the DPH website. At the county level, children were stratified by BLLs above or below the value of 5 μg/dL from 2013 to 2018. From 1998 to 2012, the county-level BLLs were striated as 10–19 μg/dL and greater than or equal to 20 μg/dL. At the ZIP code level, the data contained counts of children with BLLs below 5 μg/dL and equal to or above 5 μg/dL from 1998–2018. Due to privacy policies, any count of five or fewer children in a given geographic area is suppressed. When possible, missing values were calculated as the difference between the total children screened and the complimentary strata if that value would not be directly reported or otherwise pose a risk of identifying individuals.

All demographic data (including population counts, median household income, income to poverty ratio, and housing age) were retrieved from the US Census Bureau. Demographic data for the years 2000 and 2010 came from the decennial census. Otherwise, data were retrieved from the appropriate 5-year American Community Survey (ACS)^40^. Only the decennial census includes estimates of the number of children by single-year ages, so the proportion of children screened by age was only calculated for the years 2000 and 2010. All data were accessed via the Census API and analyzed in R^41^. Median household income values were adjusted for inflation to be in 2018 USD. These adjustments were made according to the formula in the 2018 ACS General Handbook with the updated Consumer Price Index Research Series Using Current Methods for All items^42,43^.

All maps were created using R and the open source mapping library Leaflet. The geographic boundaries used for mapping were retrieved as shapefiles from the US Census Bureau’s TIGER/Line Shapefile web interface^44^. For each map, the appropriate geographic boundaries were retrieved for the year of the data plotted. All geographic comparisons and overlaps were made according to the US Department of Housing and Urban Development United States Postal Service (HUD-USPS) ZIP Code Crosswalk Files^45^.

For analysis of ZIP code data, all ACS and census data were retrieved for 5-digit ZIP code tabulation areas (ZCTAs) which the Census Bureau defines as “generalized areal representations of United States Postal Service ZIP Code service areas”^46^. To analyze the BLL data that the DPH reports by ZIP code, each ZIP codes was matched with ACS data for the corresponding ZCTA. The ACS began in 2005, but 2011 5-year estimates were the first available at the ZCTA level. The greater Atlanta area was defined based on the ZIP codes that loosely fall within the circle formed by highway 285 (see appendix for Table S1 and Figures S1 and S2). The census tracts that correspond to this list of ZIP codes (based on HUD-USPS Crosswalk Files) were used to define the greater Atlanta area at the census tract level.

To assess the effectiveness of targeted blood lead screening, the income to poverty level ratio (IPR) was used as a measure of relative poverty. IPRs are calculated as income before taxes in the past 12 months divided by the poverty threshold which the Census Bureau assigns (in inflation-adjusted US dollars) based on the size and age of family members. The US Census Bureau reports IPRs in discrete buckets (e.g. households with IPRs from 1.00 to 1.24). Like past research, the proportion of a population with an IPR over 1.25 was used as a proxy measure for a ZCTA’s relative poverty^47^. Since higher IPRs indicate more income above the poverty level, this proportion is negatively associated with relative poverty. For example, as there is more relative poverty in a given area there is a lower proportion of people with IPRs over 1.25.

Assessing housing age is also important for understanding lead exposure risk because older homes are more likely to contain lead-based paint. Here, the proportion of houses built before 1950 was used as the measure of older housing. This proportion was calculated using the ACS estimate of the number of housing units built before 1950 divided by the total housing units.

The blood lead screening rate for every ZCTA was calculated as the total number of children screened in a ZIP code according to DPH BLL data divided by the ACS estimate of the total number of children under 6 years of age in the corresponding ZCTA. The Kruskal–Wallis test was used to test the association between two key risk factors (relative poverty and housing) and ZCTA screening rates. To assess the simultaneous effects of these factors, a linear regression was run for screening rates on the proportion of houses built before 1950 and the proportion of people with IPRs above 1.25. Additional linear regressions for screening rate that included racial indicators (e.g. the proportion of the population that is non-Hispanic black) were also evaluated.

Finally, a simple priority screening index ranging from 2–8 like the one used by Vaidyanathan et al. ^36^ was created. This index was primarily applied to census tracts as they allow intra-ZCTA analysis of small, specific areas. For comparison, the indexing system was also applied to Georgia ZCTAs and counties. When this index is created for any given year, each index value is the sum of the geographic area’s risk score for housing age and relative poverty based on that year’s data. Here, the index was applied to the most recent year with data available: 2018. The housing age risk score was assigned based on the quantile breaks in the proportion of housing built before 1950 (i.e. 0th to 25th percentile = housing risk of 1, 25th to 50th percentile = 2, 50th to 75th percentile = 3, and 75th to 100th percentile = 4). The relative poverty risk score was assigned based on quantile breaks in the proportion of the population with an IPR above 1.25. However, these poverty risk scores were reversed because lower proportions indicate more poverty and higher risk (i.e. 0th to 25th percentile = housing risk of 4, 25^th^ to 50^th^ percentile = 3, 50th to 75th percentile = 2, and 75th to 100th percentile = 1). Since both of these values are based on quantile breaks, a given area’s risk scores (and, therefore, overall priority screening index value) is dependent on the reference universe of data used to calculate the index. This same index was also applied to census tracts across the state of Georgia and the entire US to identify tracts that consistently have the highest priority screening index values.

## Results

Between 1998 and 2018, DPH reported a total of 1,657,269 blood lead tests. The total number of children screened in a given year ranged between 17,634 to 127,749. The number of children screened per year has varied widely (mean = 78,917; standard deviation = 39,278) but has generally increased over time (see Figure S3). Halfway through this time period, there was a large jump when the total number of children screened nearly doubled from 64,914 in 2008 to 122,606 in 2009. The proportion of children with BLLs above 5 μg/dL ranged from 39% (in 1998) to 1.1% (2018).

**Figure 1:**
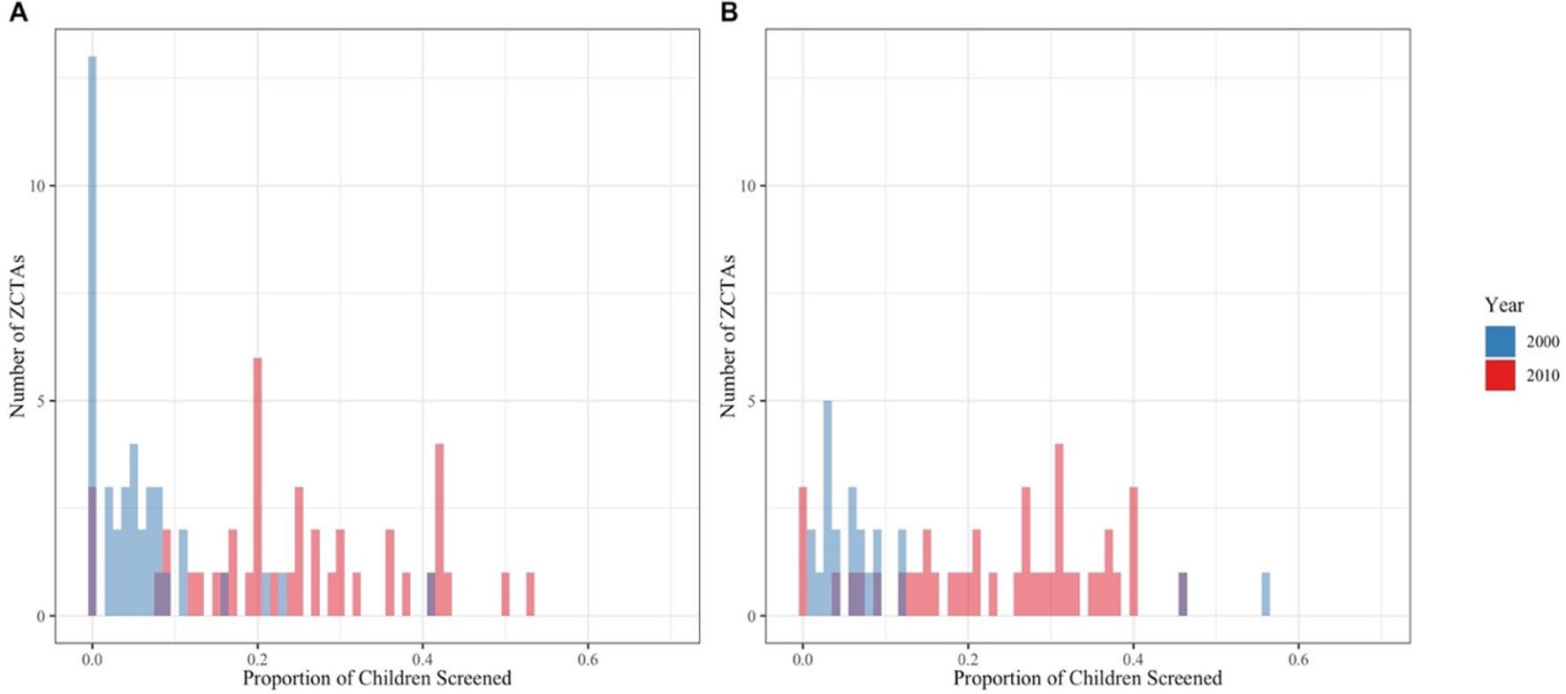
Proportion of Children Screened in Atlanta ZCTAs in 2000 and 2010 for (**A**) 0–12 Month Olds and (**B**) 12–24 Month Olds.

While all of the children screened were under six years old, every year at least 49.7% of the screened children were under two years old and at least 65.2% were under three years old. Figure 1 shows the proportions of children 0–12 and 12–24 months old that were screened in Atlanta ZCTAs in the last two census years. In both age groups, the distribution of these proportions was far more right skewed in 2000 compared to 2010. Neither age group came close to complete screening in either year. The maximum percentage of children screened in a ZCTA was 53% for 0–12 month-old children (ZCTA 30329 in 2010) and 56% for 12–24 month-old children (ZCTA 30315 in the year 2000). For racial analysis, BLL observations are striated by DPH into four groups: black, white, other, and unknown. The statewide proportion of children screened who were black ranged from 1.6% in 1998 to 37% in 2005. The proportion of screened children who were white ranged from 0.005% in 1998 to 47.5% in 2005.

**Figure 2:**
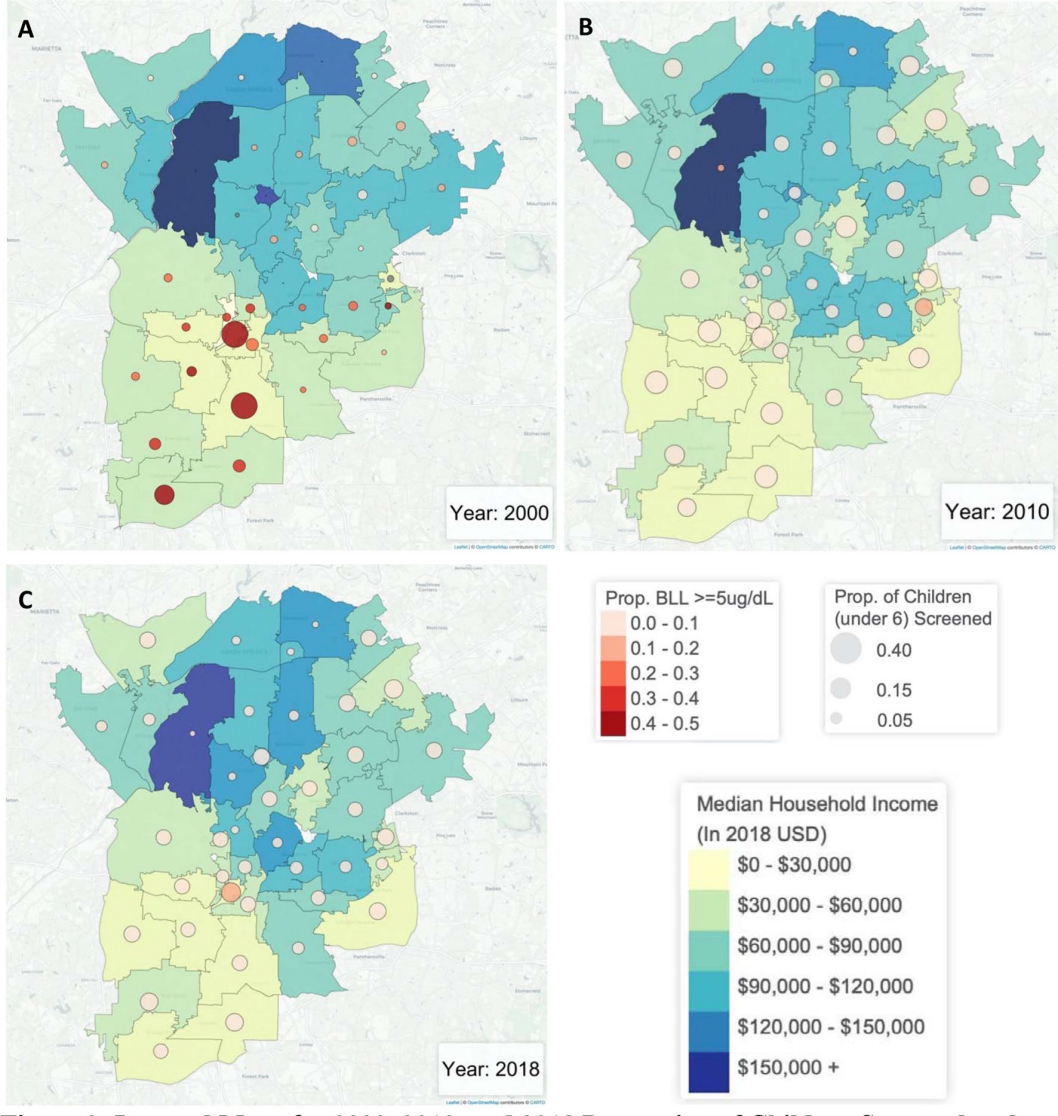
Layered Maps for 2000, 2010, and 2018 Proportion of Children Screened and Proportion of Screened Children with Blood Lead Levels at or above 5 μg/dL in Greater Atlanta Area ZCTAs by Median Household Income *Maps produced in R (version 3.6.2) using Leaflet*.

Overall, 72,570 (4.4%) of screened children were missing a ZIP code and, therefore, excluded from all ZCTA-level analyses. In the period when ZCTA-level ACS data were available (2011 to 2018), the proportion of children screened in ZCTAs ranged from 0.99% to 66.7% with a median of 12.7%. The ZCTA-level proportion of screened children with BLLs above 5 μg/dL ranged from 0 to 13.3% with a median of 1.5%. To visualize these changes over time, Fig. 2 shows a series of maps illustrating the proportion of children screened and the proportion with BLLs above 5 μg/dL. These values are layered on top of median household income to demonstrate the connection between lead exposure and wealth. Figure 2 shows these maps for the most recent year with data available (2018) and the two most recent census years (2000 and 2010). Overall, these maps reflect that the proportion of screened children with BLLs above 5 μg/dL has generally decreased while the proportion of children screened has stabilized.

From 2011 to 2018, the proportion of screened children with BLLs at or above 5 μg/dL in Atlanta ZCTAs was positively correlated with the proportion of housing built before 1950 (Pearson correlation = 0.17; *p* value = 0.0017) and negatively associated with the proportion of people with IPRs over 1.25 (Pearson correlation = − 0.15; *p* value = 0.0050). In those same years, blood lead screening rates were not significantly associated with increasing proportions of housing built before 1950 (Kruskal–Wallis *p* value = 0.4077). However, screening rates were significantly associated with ZCTAs’ quantile score for proportion of people with IPRs over 1.25 (Kruskal–Wallis *p* value =  < 2.2 × 10^–16^). A linear regression to assess the simultaneous contributions of these risk factors indicated that housing age continues to be insignificant, while relative poverty is still statistically significantly associated with screening rates when housing age is accounted for (see Appendix for Table S2). We also created two additional regression models that each assesses screening rates by housing age, relative poverty, and a racial indicator (Table S2). The proportion of housing built before 1950 was not significantly associated with the proportion screened in any model, however all of the models have a modest adjusted R^2^. These findings are consistent with the findings by Vaidyanathan et al.^36^ that poverty (in their case, WIC enrollment) but not housing age is significantly associated with screening rates when these factors are assessed individually or together in a linear regression.

**Figure 3:**
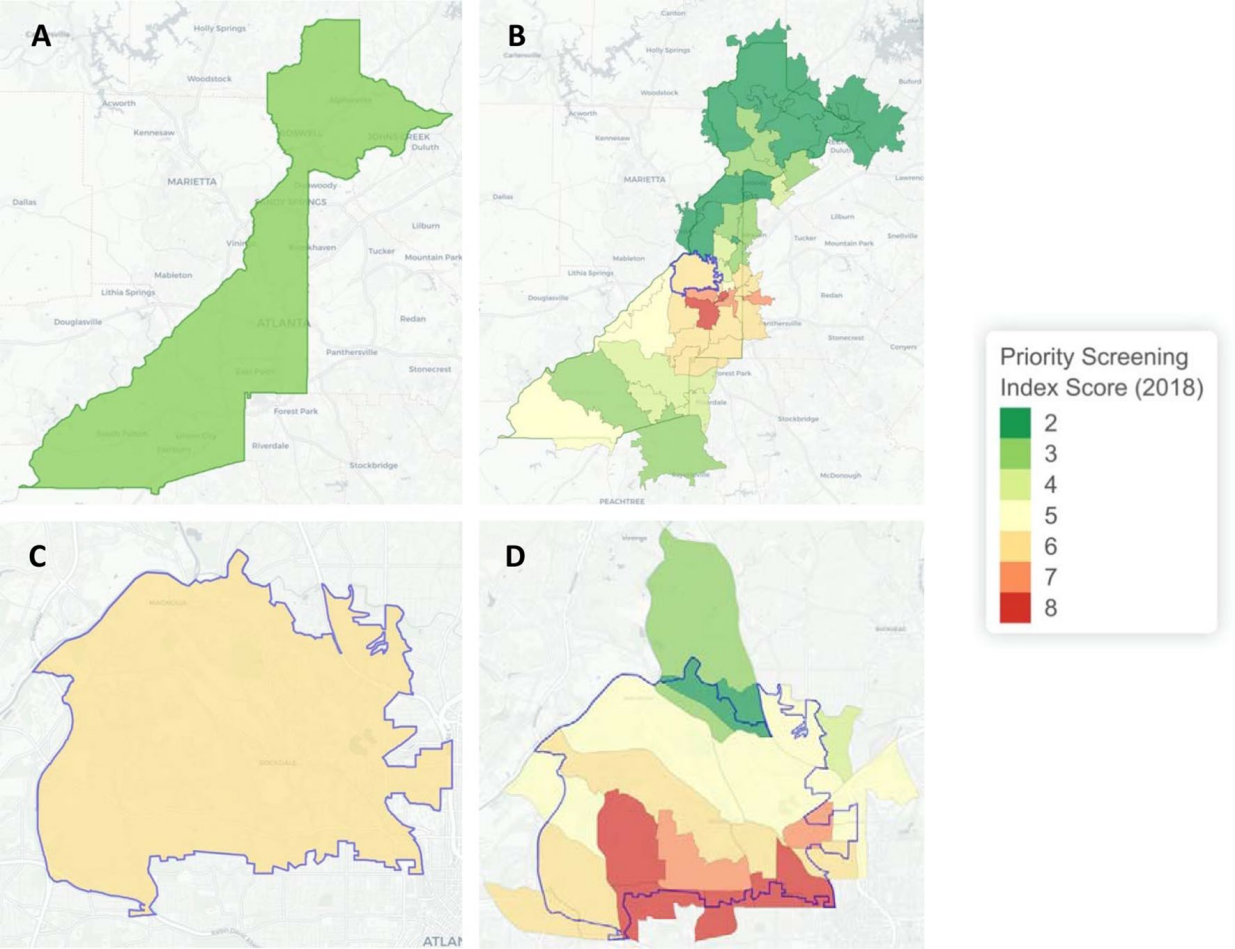
Hierarchical Priority Screening Index Values for (**A**) Fulton County, (**B**) ZCTAs in Fulton County, (**C**) ZCTA 30318, and (**D**) Census Tracts in ZCTA 30318. *Maps produced in R (version 3.6.2) using Leaflet*.

When applied to 2013 county level data, the priority screening index created here did not assign any of the 14 high risk counties the highest index value of 8. Only one (Troup County) of the counties identified by Rustin et al.^38^ was assigned an index value of 7 (see Figure S5). A key advantage of creating a priority screening index is that it is not just limited to counties, which are large geographic areas that may contain smaller areas with distinctly different lead exposure risk. Here, this priority screening index was applied to counties, ZCTAs, and census tracts. Figure 3 shows the priority screening index values of Fulton County, the most populous county in Georgia, and its underlying areas all using the state of Georgia as the reference universe. Panel A shows that when the index is applied at the county level, Fulton County is assigned the second-lowest index value of 3. However, panels B, C, and D show that underneath this value the county is quite heterogeneous. The different ZCTAs across the county (panel B) and the even finer census tracts within ZCTA 30318 (panels C and D, highlighted in panel B) reveal pockets of high-risk areas that were not reflected at the county level.

**Figure 4:**
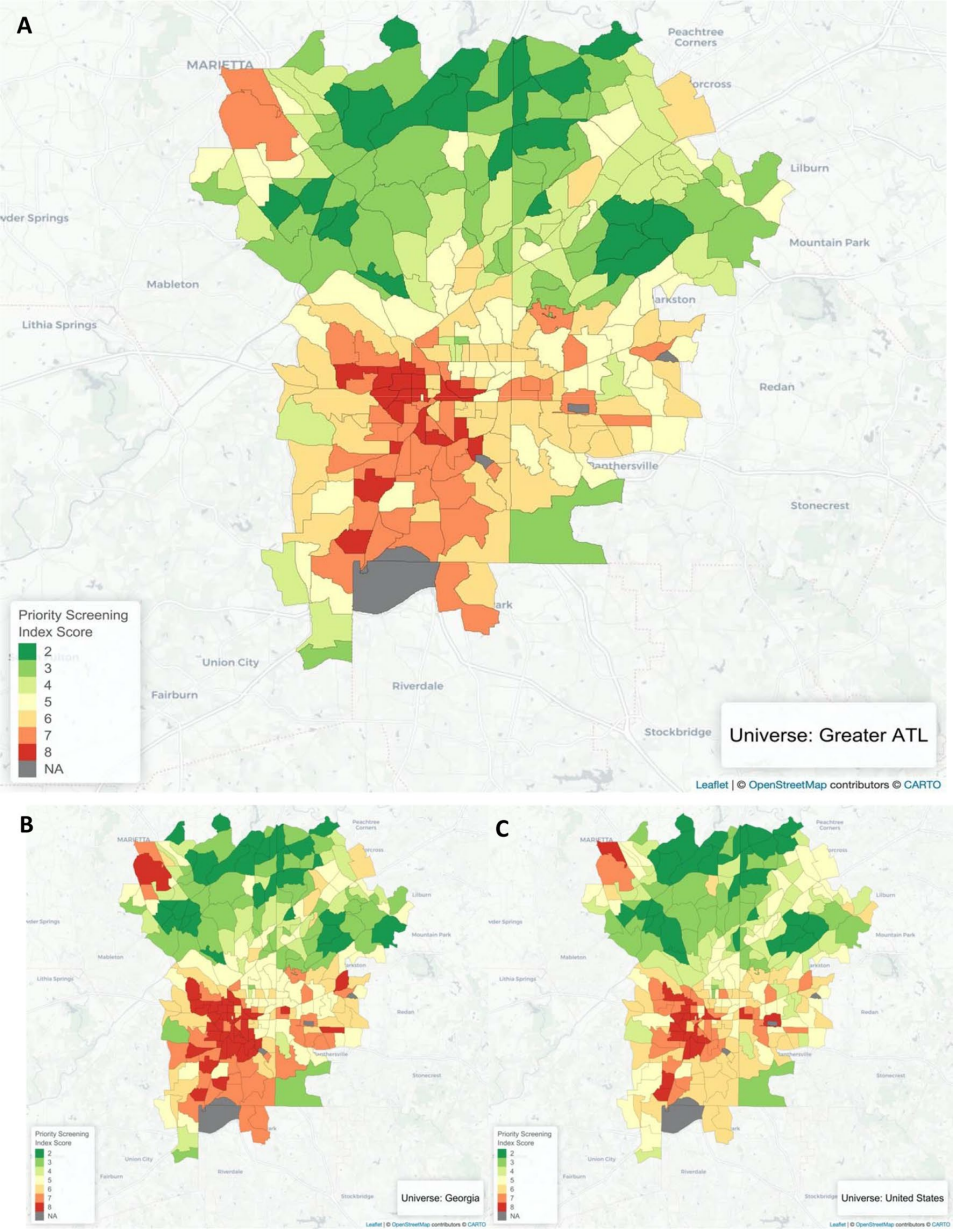
Priority Screening Index for Greater Atlanta Area Census Tracts in 2018 when the Reference Universe is (**A**) Greater Atlanta, (**B**) Georgia, and (**C**) the United States. *Maps produced in R (version 3.6.2) using Leaflet*.

Of a total 288 census tracts, 18 were given the highest priority index value of 8 across the greater Atlanta area (Fig. 4a). Since priority screening index values can change depending on the universe the index is applied to, recalculating the index based on different reference universes is one way to evaluate results. Figure 4 shows the priority screening index values of Atlanta area census tracts in 2018 as calculated when the reference universe is the greater Atlanta area (panel A), Georgia (panel B), and the entire US (panel C). While some Atlanta census tracts show increasingly lower PSI values, across every universe there is a consistent cluster of high-risk census tracts in the westside of Atlanta (Fig. 4). These nine tracts in the westside of Atlanta continues to have the highest priority values (7 and 8), suggesting that screening this area is of the highest priority.

**Figure 5:**
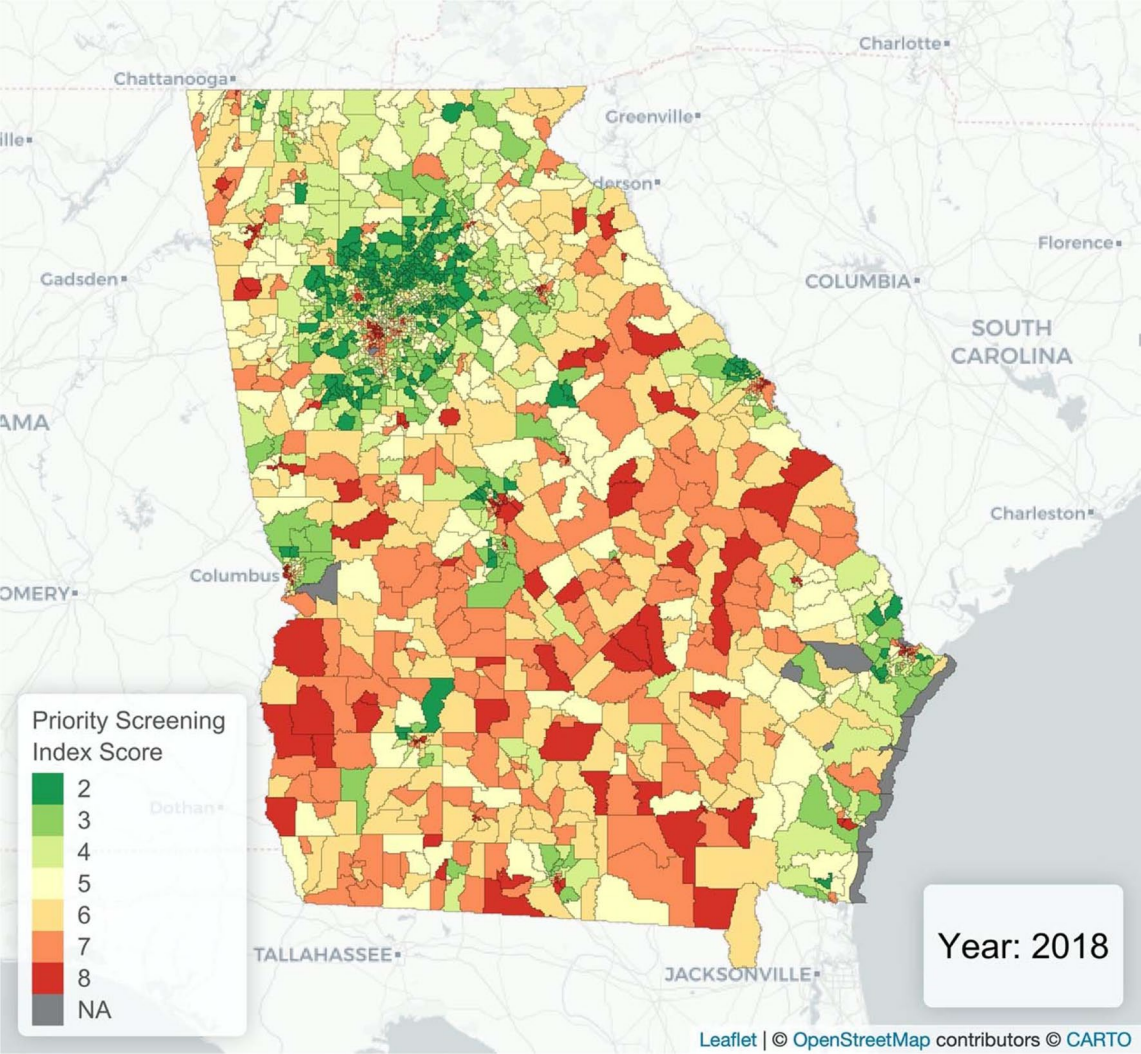
Priority Screening Index for Georgia Census Tracts when Georgia is the Reference Universe. *Map produced in R (version 3.6.2) using Leaflet*.

Some areas outside of Atlanta with clusters of high-risk tracts include western Savannah, southeastern Macon, and central Athens (Fig. 5), with the state as the reference universe. In addition to these urban areas, there are also high priority census tracts along the southern section of the Georgia-Alabama border.

## Discussion

Blood lead screening has generally been increasing in the Greater Atlanta area since 1998. However, as the CDC has stressed, effectively targeting those screening tests is crucial to identify the most children at risk of lead exposure^21^. The lack of significant association between ZCTA screening rates and the proportion of houses built before 1950 is concerning because the lead paint often found in older housing is a major source of lead exposure^39,48^. This lack of association is especially troubling because it has persisted for years despite being highlighted by Vaidyanathan et al.^36^. In 2018, 16.5% of the total 517,488 housing units in the greater Atlanta area were built before 1950. Therefore, children in more than 85,000 houses could have increased risk for lead exposure without being effectively targeted for blood lead screening.

The results of this priority screening index cannot be directly compared to the results of the Vaidyanathan et al.^36^ because of the difference in geographic scale (census tracts versus neighborhoods). Additionally, that paper was primarily focused with the development of a neighborhood-level spatial strategy rather than the results of applying the screening index to Atlanta neighborhoods. However, the authors did note that neighborhoods in the south of Atlanta tended to have lower incomes, while neighborhoods in east central Atlanta had the highest proportion of older homes. Here, many tracts in southern Atlanta did have higher priority screening index scores which is consistent with a lack of wealth in that area. Areas in east central Atlanta did continue to have high proportions of older homes. However, census tracts in this area tended to have only mid-level index scores as the risk was mitigated by relative wealth outweighing the effect of these older homes. This could be an effect of demographic changes in the area as Atlanta has been identified as the fourth most gentrifying central city in the nation^49^. Furthermore, a map released by the City of Atlanta’s Department of Planning and Community Development identified every neighborhood in central and eastern Atlanta as being under some gentrification pressure between the susceptible and mature stages^50^.

The most recent assessment of areas in Georgia with high lead exposure risk is the map of high-risk counties from Rustin et al.^38^. The counties highlighted in that paper have little correspondence with the counties identified as high risk by this priority screening index (see Figure S5). The Rustin et al.^38^ map may therefore not be capturing risk related to housing age and relative poverty. Instead, this prevalence-based map may reflect population size and screening rates more than an increased risk of exposure.

Focusing in on small geographic areas is important to identify those at risk for the more subtle low-level lead exposures facing children today. The priority screening index calculated here focused on census tracts because they are a small geographic area with available census bureau data. As the hierarchical maps demonstrate, counties and ZCTAs with a low- or mid-level priority screening index value can contain smaller regions of high-priority areas that might not be targeted for screening under a county-level strategy.

The priority screening index for census tracts in the greater Atlanta area shows a cluster of high priority tracts in the westside of Atlanta. This area includes English Avenue and Vine City, historically black neighborhoods that have been highlighted in the media for incredibly high poverty and unemployment rates^51–53^. While this area was identified as high risk here based on housing age and relative poverty, industrial slag (another source of lead) has also been found in that area^54,55^. This result is further strengthened by the consistently high index values assigned to these tracts when the index is applied to the state of Georgia and the entire nation. This easy way to run a preliminary check on the strength of results is a key advantage of this index for anyone who might implement it.

While easier to replicate and useful for targeting, analyzing BLLs and screening rates by geographic area is inherently less precise than analyzing address-level data. Here, data by individual screened children was not available due to HIPAA protections which limits the precision of this analysis. The lack of individual address data also prevented BLL and screening data from being aggregated to different geographic areas (other than ZCTAs and counties) so census tract level screening rates could not be assessed.

Similar to the housing data used by Vaidyanathan et al.^36^, the housing data used here did not have any information on lead remediation or other interventions to eliminate sources of lead. Older houses may no longer pose an exposure risk if they have been successfully treated to remove lead so some areas with a high proportion of older houses may pose a lower risk than the priority screening index indicates. Additionally, housing age and all other demographic data were estimates from the ACS, but the variation around these estimates was not incorporated into the priority screening index. The ACS data only exist for ZCTAs from 2011 and 2018 which limited the years that could be included in some analyses. Since there is no safe level of lead exposure for children, distinctions about priority screening and treatment are necessarily relative like the CDC’s 97.5^th^ reference value. Because this index is also based on percentiles, it is inherently sensitive to scale. The index score of any given place will be dependent on the geographic level (e.g. census tracts, ZCTAs, counties) of the data being used. Finally, other sources of lead such as coal ash, air pollution, and contaminated food, soil, and water were not included in the priority screening index which limits its ability to capture lead exposure risk. These factors can also be major contributors to lead exposure and further work should be done to assess, incorporating such sources into the priority screening indexes^6,56–59^.

There are many other opportunities for future work to continue to assess the risk of low-level lead exposure in Atlanta. First, analysis of address-level data could help build an understanding of blood lead screening rates. Using individual data, screening tests and results could be aggregated at the census tract level and the relationship between census tract screening rates and housing age and relative poverty could then be assessed. This higher resolution data would also allow for comparisons between census tract level priority screening index values and screening rates. These differences can then be used to identify specific under-screened census tracts to be targeted for increased screening (see Figure S6 for difference scores at the larger ZCTA level).

As more surveillance data about low-level lead exposure become available, it will also be important to continue to assess lead exposure risk factors. Identifying socioeconomic factors associated with low but elevated BLLs is crucial to help target blood lead screening. This work could help validate this priority screening index or potentially highlight other factors besides housing age and relative poverty that should be incorporated into the index.

Effectively identifying children who are more likely to be exposed to lead is especially critical to end chronic lead exposure. Identifying these children may be difficult with lower public concern and a need for highly specific targeting. However, this work is crucial as the danger of lead exposure is far from over and the remaining risk of exposure is not equally distributed. To continue decades of work and end racial and socioeconomic disparities, public health officials need to focus more than ever on targeting specific high-risk areas for blood lead screening and eventual intervention.

## Supplementary information

Supplementary Information.
