# Supplementary material for: A new screening index to better target low-level lead exposure in Atlanta, Georgia
Source: Sci Rep. 2020 Oct 22;10:18087. doi: 10.1038/s41598-020-75000-0 (PMC7581719; doi:10.1038/s41598-020-75000-0)
Supplement: Supplementary file 1 — Supplementary Information. [file 41598_2020_75000_MOESM1_ESM.docx]

**APPENDIX**


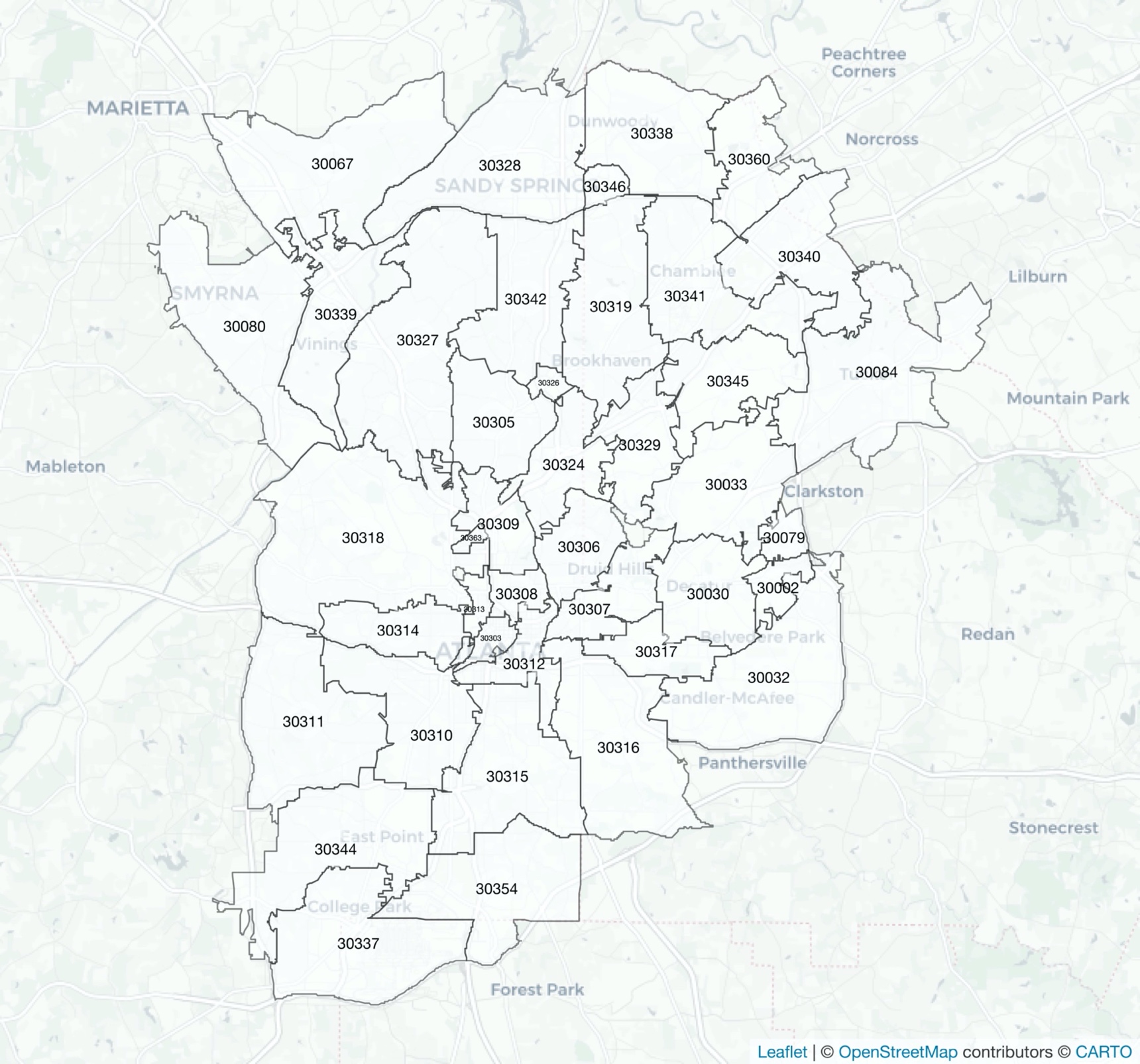


**Figure S1: Map of All ZCTAs Defined as the Greater Atlanta Area. *Map produced in R (version 3.6.2) using*** [***Leaflet***](https://rstudio.github.io/leaflet/)

**Table S1: ZCTAs Defined as the Greater Atlanta Area**

**
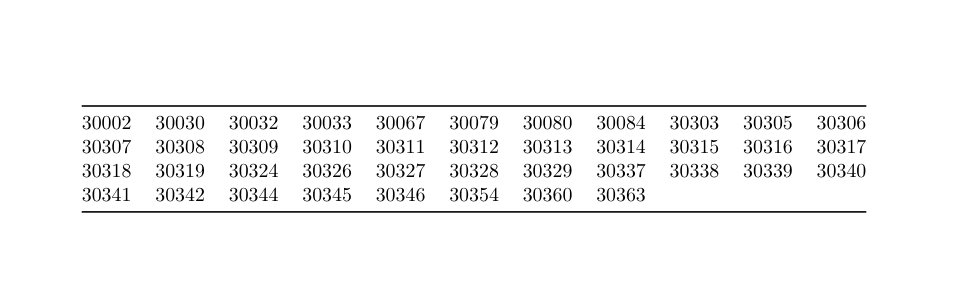
**


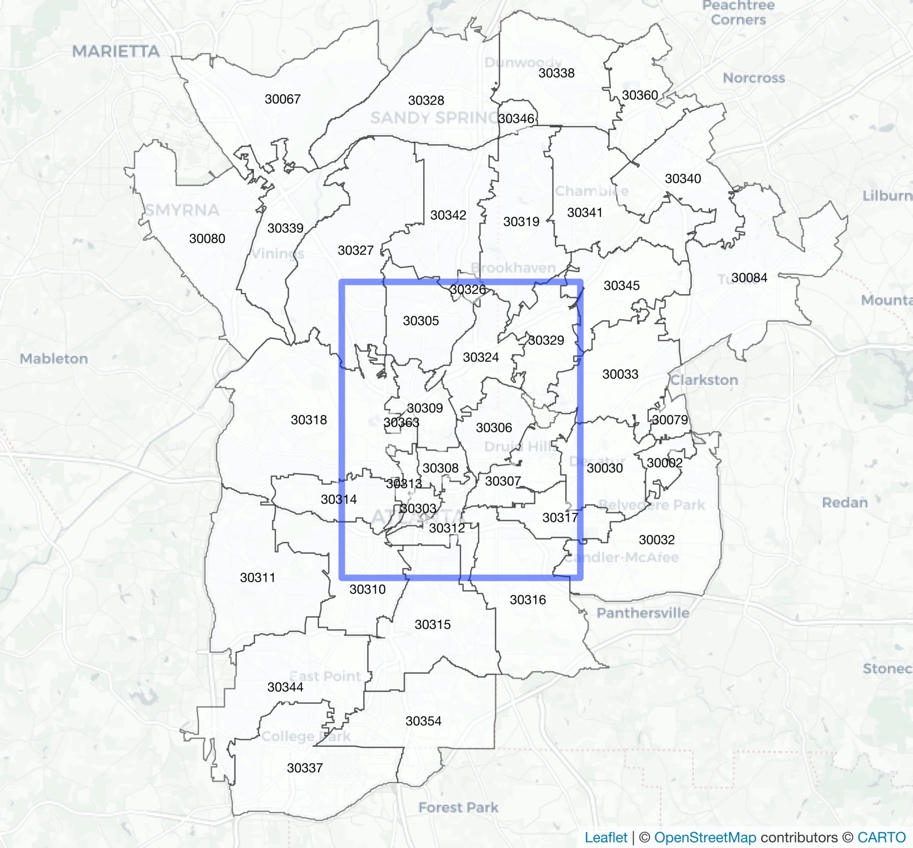

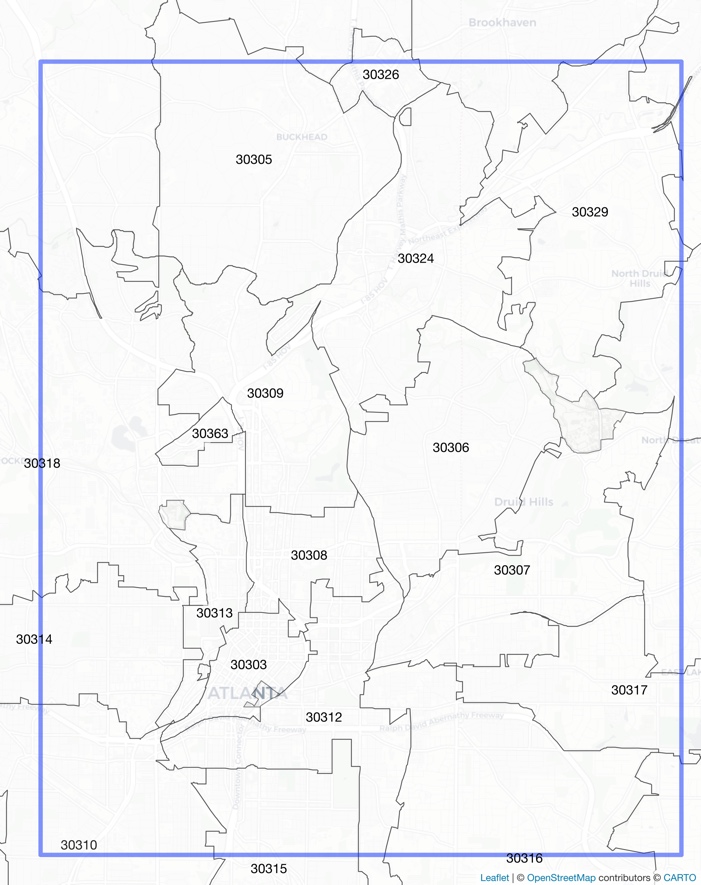


**A**

**B**

**Figure S2: Map of (A) All ZCTAs Defined as the Greater Atlanta Area and (B) a Close-Up View of ZCTAs in Central Atlanta. *Maps produced in R (version 3.6.2) using*** [***Leaflet***](https://rstudio.github.io/leaflet/)

**
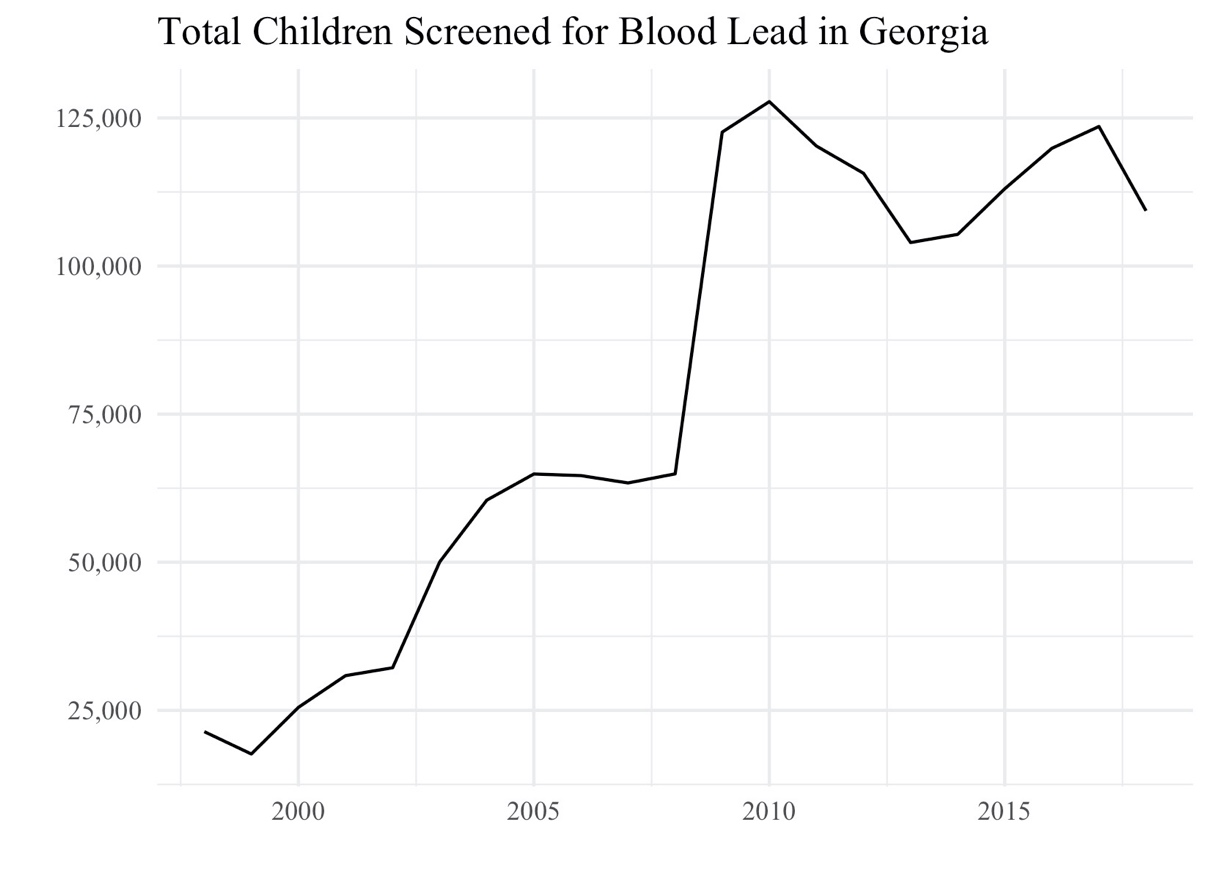
**

**Figure S3: Total Children Screened for Blood Lead in Georgia, 1998-2018**


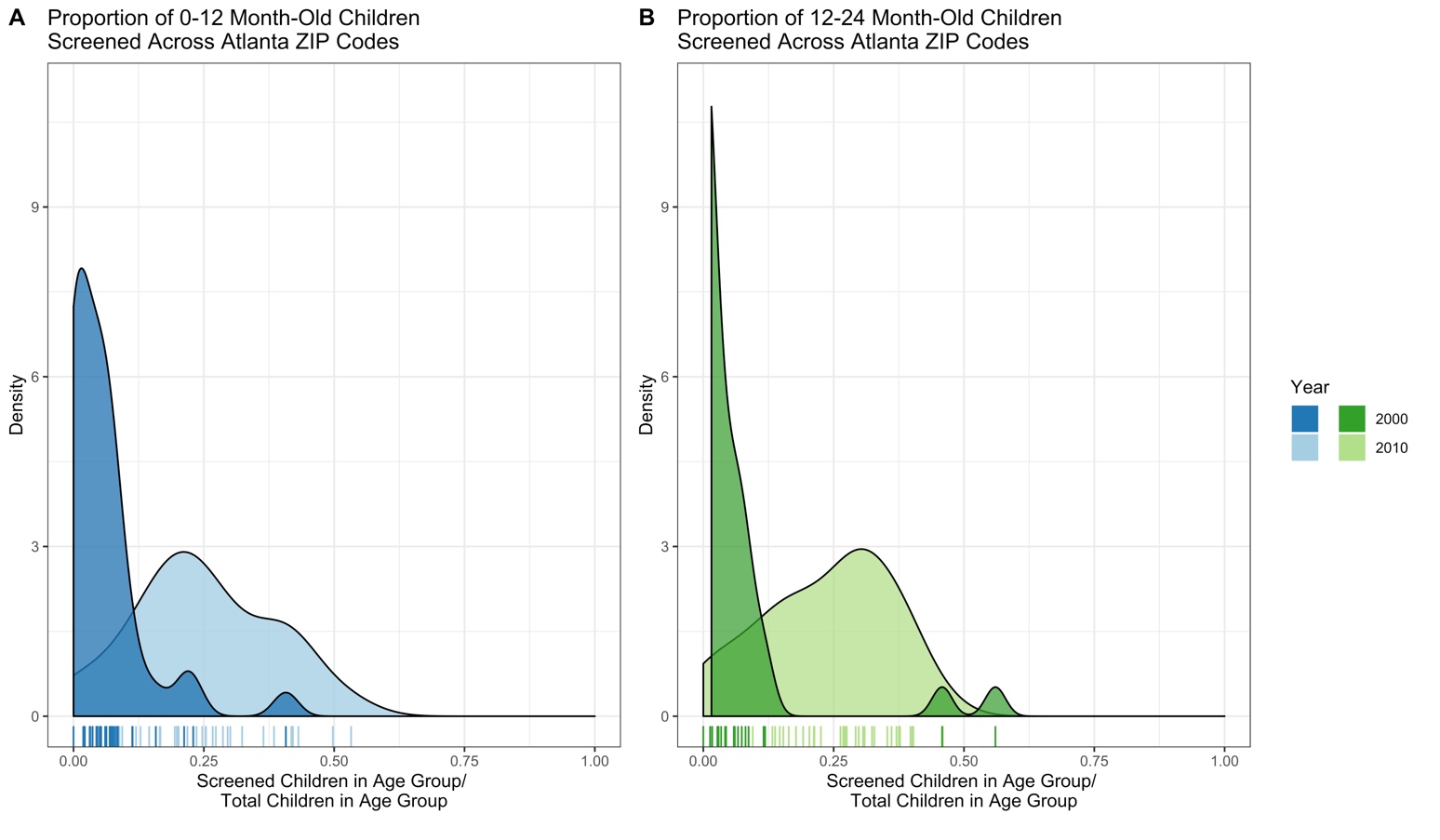


**Figure S4: Raw Density Distribution of the Proportion of Children Screened in 2000 and 2010 for (A) 0-12 Month Olds and (B) 12-24 Month Olds**

**
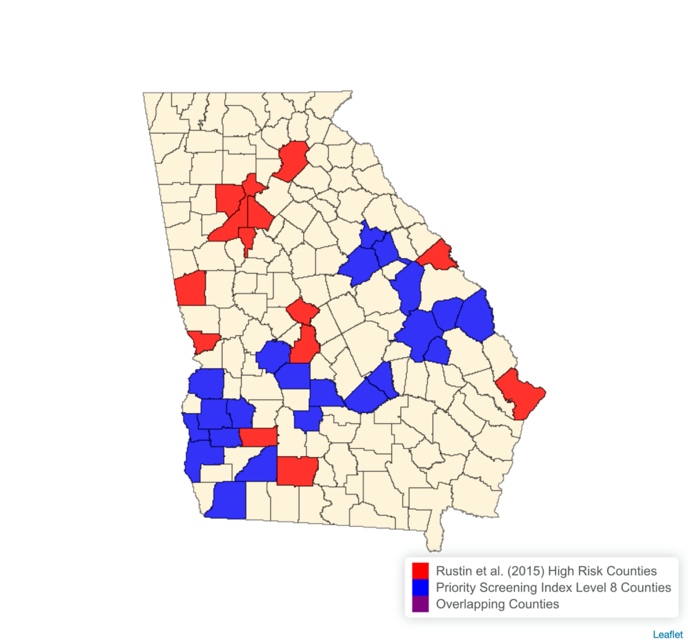

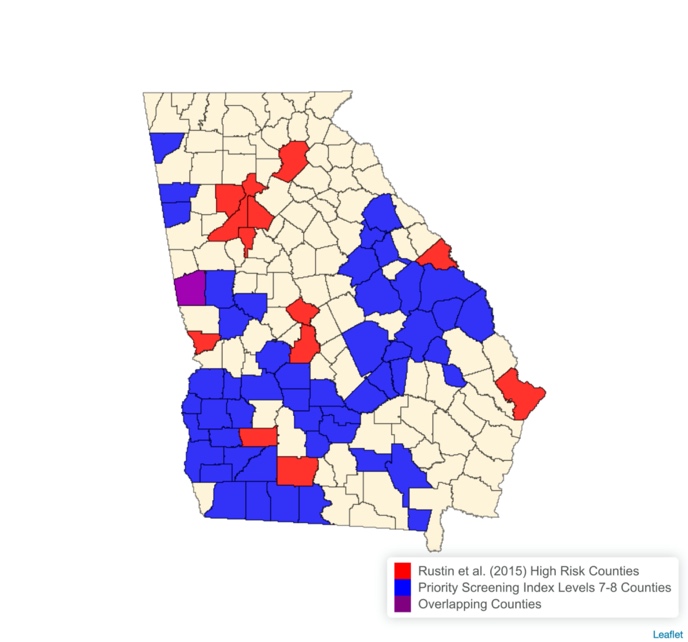
**

**B**

**A**

**Figure S5: Comparison of Counties Identified as “High Risk” by Rustin et al. (2015) and Counties Identified By the Priority Screening Index as (A) Level 8 and (B) Levels 7 and 8 When Georgia is the Reference Universe. *Maps produced in R (version 3.6.2) using*** [***Leaflet***](https://rstudio.github.io/leaflet/)

**Table S2: Linear Regression Models for the Proportion of Children Screened in Greater Atlanta ZCTAs**


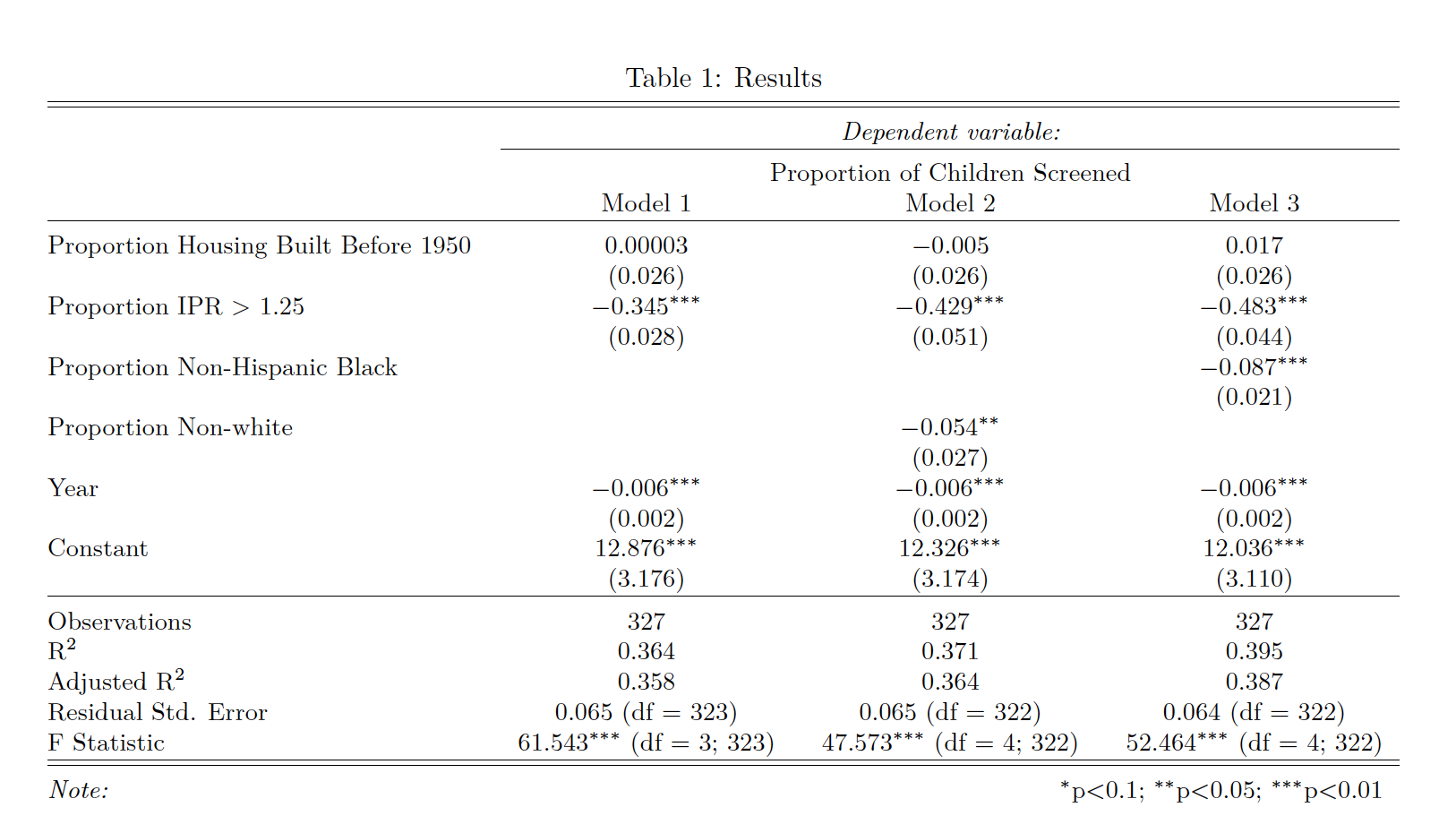


**
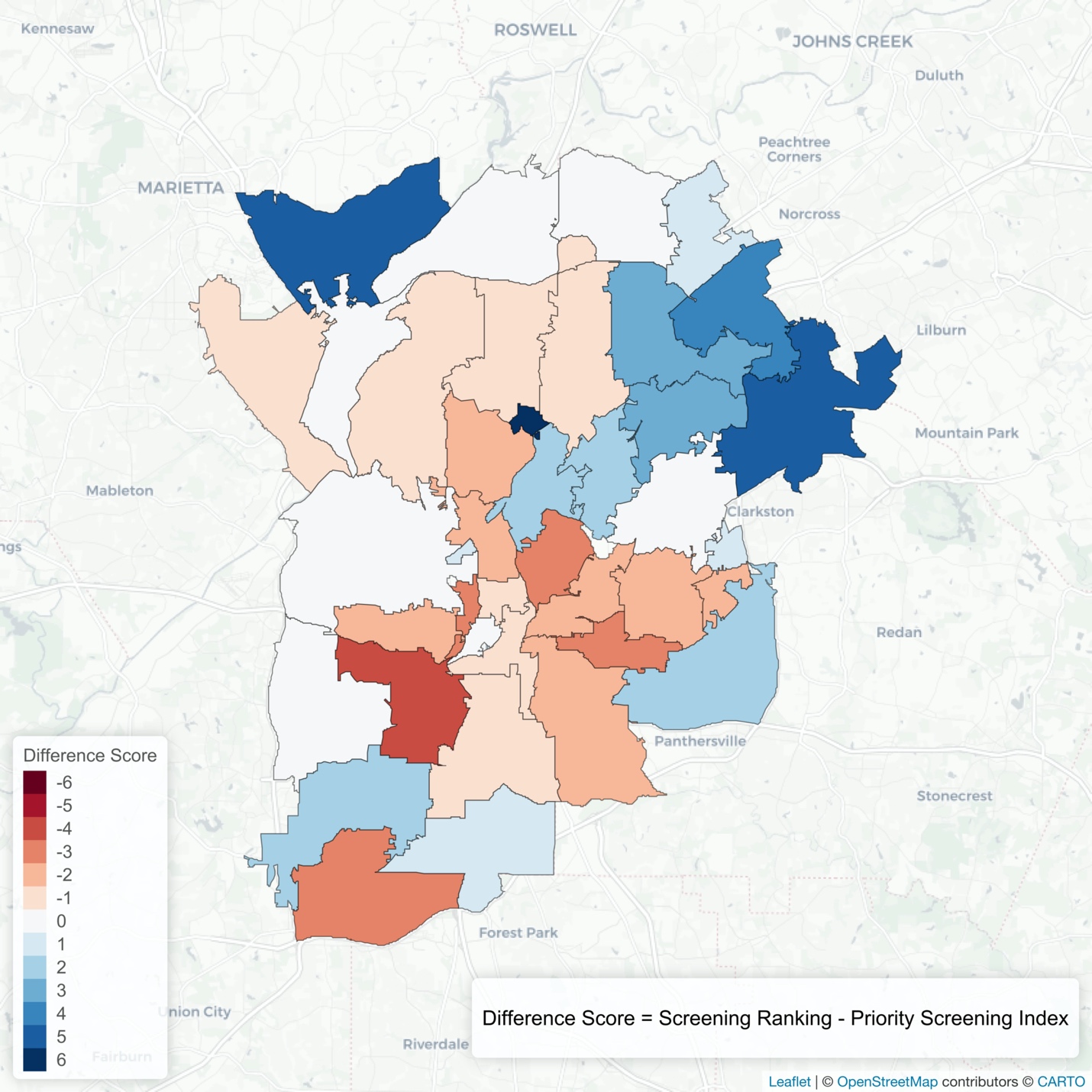
Figure S6: Greater Atlanta Area ZCTAs Difference Scores. *Map produced in R (version 3.6.2) using*** [***Leaflet***](https://rstudio.github.io/leaflet/)
